# A reliable mixed-integer linear programming formulation for data-driven model predictive control in buildings

**DOI:** 10.1016/j.mex.2025.103470

**Published:** 2025-07-01

**Authors:** Peter Klanatsky, François Veynandt, Christian Heschl

**Affiliations:** Hochschule Burgenland University of Applied Sciences, Campus Pinkafeld, Steinamangerstraße 21, 7423 Pinkafeld, Austria

**Keywords:** Building energy management, Model predictive control, Data-driven predictive control, Thermally activated building structure, Smart shading control, Data-driven building modelling, Optimization algorithm, Optimization problem formulation with Mixed-Integer Linear Programming for Data-driven Model Predictive Control of buildings with Thermally Activated Building Structures and shading system

## Abstract

Integrating renewable energy sources into buildings requires advanced control strategies to enhance demand-side flexibility. Data-driven Model Predictive Control (DMPC) has shown significant promise in this area. Buildings with Thermally Activated Building Structures (TABS) and glass façade present flexibility potential, but have a challenging thermal balance, due to high thermal inertia and significant solar gains. In such buildings, a DMPC jointly controlling TABS and the shading system is advantageous. However, the only known implementations enabling this combination rely on a white-box model, limiting the replicability of the approach due to the required modelling effort. To facilitate the implementation of a DMPC for such a combined control task, this paper presents in details a suitable optimisation algorithm:

• specifically designed for buildings with TABS and shading systems,
• based on a grey-box model of thermal zones (a reduced order state-space model),
• using a reliable and efficient Mixed-Integer Linear Programming (MILP) formulation,
• handling thermal comfort as constraints, avoiding weighting factors in the objective function.

## Graphical abstract

**Figure:**
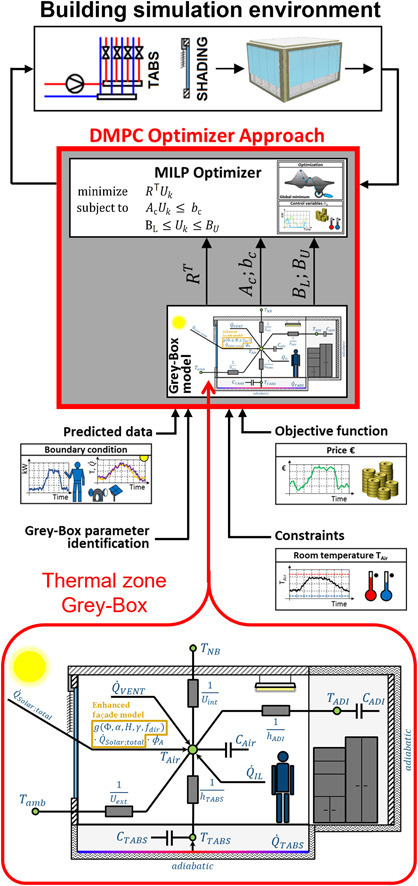

Specifications table

**Table:** 

Subject area:	Energy
More specific subject area:	Optimization algorithm for data-driven model predictive control of buildings for efficient and flexible energy management
Name of your method:	Optimization problem formulation with Mixed-Integer Linear Programming for Data-driven Model Predictive Control of buildings with Thermally Activated Building Structures and shading system
Name and reference of original method:	The Mixed-Integer Linear Programming algorithm is a well-established method [1], which is employed here with a simplified physical model (grey-box) of a building thermal zone including a special façade model, as described in [2].
Resource availability:	*-*

## Background

Demand Side Management (DSM) plays a strategic role in maintaining power grid balance as the share of variable renewable energy increases [3]. The integration of renewable energy sources into buildings increasingly relies on advanced control strategies to enhance demand-side flexibility [4]. Among these, Model Predictive Control (MPC) has emerged as a promising approach for building energy management [5,6]. MPC optimizes a system control by predicting future building states, including forecast data such as weather [7], occupancy [8], energy demand [9], and power prices [10]. This predictive capability enables MPC to improve energy efficiency and facilitate integration of renewable energy sources [11,12]. Relying on a prediction model, MPC solves an optimisation problem with an objective function and constraints [13].

A data-driven variant, Data-driven Model Predictive Control (DMPC), has shown potential for managing complex building systems [14]. DMPC leverages measured data and numerical optimization to identify system parameters efficiently. It often incorporate thermal comfort constraints as a term in the objective function [[15], [16], [17]], requiring manual tuning of weighting factors to balance energy costs and comfort penalties. Treating thermal comfort as a bound constraint instead eliminates the need for weighting factors, enhancing reproducibility. By directly constraining indoor temperatures within a predefined comfort range, the solution space is reduced, enabling more efficient and reliable optimization [18].

This work focuses on buildings with Thermally Activated Building Structures (TABS) and glass façades equipped with shading systems. TABS, integrated into floor and ceiling structures, provide heating and cooling with high thermal inertia, while the glass façade and shading system dynamically manage solar heat gains. Coordinating these systems, to balance passive solar gains with active heating and cooling, is challenging due to their differing thermal response times [[19], [20], [21], [22]]. Integrated control approaches have demonstrated energy savings by jointly controlling Heating, Ventilation and Air Conditioning (HVAC) systems and shading blinds [23,24]. However, only two implementations of MPC for the combined control of TABS and shading systems have been identified to date [25,26]. Both studies rely on detailed physical (white-box) models of the building, which hinder replicability due to significant development time.

In this context, this work presents an integrated DMPC, for controlling both the heating/cooling system and the shading system, based on a simplified physical (grey-box) model, enabling an automatic parameter identification. This grey-box state-space model is based on a reduced resistance-capacitance (RC) approach [27]. It includes a façade model for determining solar gains on a shaded glass façade through a characteristic map [28]. This model strikes a balance between computational efficiency and accuracy, requiring only two weeks of operational data for parameter identification. Additionally, handling comfort requirements as bound constraints ensures reliable and flexible optimisation, with the seamless implementation of various objectives, such as minimising energy consumption, costs or CO_2_ emissions.

The related research article [29] presents a simulation study, employing this method in a DMPC to control an office building, comparing its performance to a standard rule-based controller. Its applicability and performance have also been demonstrated over a one-year period in a real operating building [30].

## Method details

### 1 Nomenclature

**Table:** 

$A_{c}^{k}$	[…]	Matrix ($5N_{p}\times N_{u}N_{p}$) of linear coefficients for the inequality constraints, over the prediction horizon starting at time step k
$A_{cT}^{k}$	[…]	Matrix ($2N_{p}\times N_{u}N_{p}$) of linear coefficients for the inequality constraints on the room air temperature, over the prediction horizon starting at time step k
$A_{cSh1}^{k}$	[…]	Matrix ($N_{p}\times N_{u}N_{p}$) of linear coefficients for the first inequality constraint on the shading control variables, over the prediction horizon starting at time step k
$A_{cSh2}^{k}$	[…]	Matrix ($N_{p}\times N_{u}N_{p}$) of linear coefficients for the second inequality constraint on the shading control variables, over the prediction horizon starting at time step k
$A_{cSh3}^{k}$	[…]	Matrix ($N_{p}\times N_{u}N_{p}$) of linear coefficients for the third inequality constraint on the shading control variables, over the prediction horizon starting at time step k
$A_{k+i}$	[-]	Matrix ($N_{x}\times N_{x}$) of linear terms relative to the state variables $x_{k+i}$, at time step k+i
$A_{X}$	[m²]	Surface area of glass façade number X
$b_{c}^{k}$	[…]	Vector ($5N_{p}\times \;1$) of the right-hand side coefficients for the inequality constraints, over the prediction horizon starting at time step k
$b_{cT}^{k}$	[°C]	Vector ($2N_{p}\times \;1$) of the right-hand side coefficients for the inequality constraints on the room air temperature, over the prediction horizon starting at time step k
$b_{cSh1}^{k}$	[-]	Vector ($N_{p}\times \;1$) of the right-hand side coefficients for the first inequality constraint on the shading control variables, over the prediction horizon starting at time step k
$b_{cSh2}^{k}$	[-]	Vector ($N_{p}\times \;1$) of the right-hand side coefficients for the second inequality constraint on the shading control variables, over the prediction horizon starting at time step k
$b_{cSh3}^{k}$	[-]	Vector ($N_{p}\times \;1$) of the right-hand side coefficients for the third inequality constraint on the shading control variables, over the prediction horizon starting at time step k
$B_{k+i}$	[°C]	Matrix ($N_{x}\times N_{u}$) of linear terms relative to the control variables $u_{k+i}$ at time step k+i
$B_{L}$	[-]	Vector ($N_{u}N_{p}\times \;1$) of the lower bounds of the control variables profile $U_{k}$
$B_{U}$	[-]	Vector ($N_{u}N_{p}\times \;1$) of the upper bounds of the control variables profile $U_{k}$
$c_{p;air}$	[J/kg/K]	Specific thermal capacity of ventilation air
C	[-]	Vector ($1\;\times N_{x}$) to extract the output variable
$C_{ADI}$	[J/K]	Thermal capacitance of internal masses (e.g., furniture), identified parameter of the grey-box model
$C_{Air}$	[J/K]	Thermal capacitance of the room air, identified parameter of the grey-box model
$C_{j}$	[J/K]	Thermal capacitance of the grey-box model (schema of Fig. 1)
$C_{TABS}$	[J/K]	Thermal capacitance of the Thermally Activated Building Structures (TABS): heating or cooling slabs, identified parameter of the grey-box model
$COP_{FH}$	[-]	Coefficient of performance of the considered reversible heat pump (heating mode)
$CTRL_{FH}^{k+i}$	[-]	Control variable $CTRL_{FH}^{k+i}\in \left[0,1\right]$ of the thermal load of the floor heating system, at time step k+i
$CTRL_{CC}^{k+i}$	[-]	Control variable $CTRL_{FH}^{k+i}\in \left[0,1\right]$ of the thermal load of the ceiling cooling system, at time step k+i
$EER_{CC}$	[-]	Energy efficiency ratio of the considered reversible heat pump (cooling mode)
$f_{dir}$	[-]	Fraction of direct irradiance in global solar irradiance, parameter of the façade model characteristic map
$F_{k}$	[-]	Matrix ($N_{p}\times N_{x}$) of the linear terms relative to the state variables $x_{k}$ at time step k, over the prediction horizon starting at time step k
$g(\phi ,\alpha ,H,\gamma ,f_{dir})$	[-]	Function representing the characteristic map, which defines the overall Solar Heat Gain Coefficient through the glass façade including shading with venetian blinds, according to the façade model
$g_{win}$	[-]	Energy transmission coefficient of the glass façade
$h_{ADI}$	[W/K]	Heat transfer coefficient, reversed value of the thermal resistance from the internal masses to the room air, identified parameter of the grey-box model
$h_{shX}^{k+i}$	[-]	Control variable $h_{shX}^{k+i}\in \left[0,1\right]$ to set the height of the external shading blinds on façade number X, at time step k+i,
$h_{shX.ph}^{k}$	[-]	Vector ($N_{p}\times \;1$) of the control variable to set the height of the external shading blinds on façade number X, over the prediction horizon starting at time step k
$h_{TABS}$	[W/K]	Heat transfer coefficient, reversed value of the thermal resistance from the Thermally Activated Building Structures (TABS, i.e., heating or cooling slabs) to the room air, identified parameter of the grey-box model
H	[-]	Shaded fraction in height of the glass façade, parameter of the façade model characteristic map
$H_{ismaxX}^{k+i}$	[-]	Binary control variable for the shading control of façade number X, indicating if the venetian blinds are fully drawn at time step k+i
$H_{ismaxX.ph}^{k}$	[-]	Vector ($N_{p}\times \;1$) of the binary control variable for the shading control of façade number X, indicating if the venetian blinds are fully drawn ($H_{ismaxX}=1$), over the prediction horizon starting at time step k
i	[-]	Time step index of the prediction horizon $i\in \{0,1,\ldots ,N_{p}-1\}$
$J_{cost}$	[€]	Result of the objective function to minimize the costs of energy
$J_{CO_{2}}$	[kgCO_2_e]	Result of the objective function to minimize the equivalent CO_2_ emissions of energy
$J_{energy}$	[J]	Result of the objective function to minimize the energy consumption
k	[-]	Current time step, initial time step of the prediction horizon
$N_{F}$	[-]	Number of external façade orientations of the thermal zone
$N_{p}$	[-]	Number of time steps of the prediction horizon
$N_{u}$	[-]	Number of control variables
$N_{x}$	[-]	Number of state variables
${\dot{q}}_{sol.B.X}^{k+i}$	[W/m²]	Specific direct solar irradiance in the plane of façade number X, at time step k+i (${\;}_{B}$ stands for beam)
${\dot{q}}_{sol.D.X}^{k+i}$	[W/m²]	Specific diffuse solar irradiance in the plane of façade number X, at time step k+i
${\dot{q}}_{sol.G.X}^{k+i}$	[W/m²]	Specific global solar irradiance in the plane of façade number X, at time step k+i
${\dot{q}}_{sol.G.cor.X}^{k+i}$	[W/m²]	Specific global solar irradiance in the plane of façade number X, at time step k+i, corrected for the different transmittance of direct and diffuse solar radiations through a glazing
${\dot{q}}_{sol.G.cor.Sh.limit.X}^{k+i}$	[W/m²]	Specific global solar irradiance in the plane of façade number X, at time step k+i, corrected for the different transmittance of direct and diffuse solar radiations through a glazing, and accounting for the shading of venetian blinds with the limit slat angle $\gamma_{limit}^{k+i}$ blocking direct solar radiation.
${\dot{Q}}_{CC}^{k+i}$	[W]	Thermal power transmitted to the zone through the ceiling cooling system, at time step k+i
${\dot{Q}}_{CC_{n}}^{k+i}$	[W]	Nominal thermal power transmitted to the zone through the ceiling cooling system (at time step k+i)
${\dot{Q}}_{FH}^{k+i}$	[W]	Thermal power transmitted to the zone through the floor heating system, at time step k+i
${\dot{Q}}_{FH_{n}}^{k+i}$	[W]	Nominal thermal power transmitted to the zone through the floor heating system (at time step k+i)
${\dot{Q}}_{IL}^{k+i}$	[W]	Thermal power from internal loads: e.g., electrical power and occupants, at time step k+i
${\dot{Q}}_{sol.in.noSh}^{k+i}$	[W]	Incoming solar irradiance, after the glass façade without shading, at time step k+i
${\dot{Q}}_{Solar;total}$	[W]	Global solar irradiance over a façade, before crossing the glass façade
${\dot{Q}}_{Solar;in}$	[W]	Global solar irradiance over a façade, after crossing the glass façade including shading, according to the façade model
${\dot{Q}}_{TABS}$	[W]	Thermal power transmitted to the zone through the Thermally Activated Building Structure (TABS, i.e., heating or cooling slabs)
${\dot{Q}}_{VENT}$	[W]	Thermal power transmitted to the zone through the mechanical ventilation system
$r_{B.X}^{k+i}$	[-]	Correction coefficient for the direct transmittance of the glass façade number X, at time step k+i
$r_{D}$	[-]	Correction coefficient for the diffuse transmittance of the glass façade, independent of the façade orientation
$R_{cost}$	[€]	Vector ($N_{p}\times \;1$) defining the objective function to minimize the costs of energy
$R_{CO_{2}}$	[kgCO_2_e]	Vector ($N_{p}\times \;1$) defining the objective function to minimize the equivalent CO_2_ emissions of energy
$R_{energy}$	[J]	Vector ($N_{p}\times \;1$) defining the objective function to minimize the energy consumption
$R_{i}$	[K/W]	Thermal resistance of the grey-box model (schema of Fig. 1)
$s_{shX}^{k+i}$	[-]	Control variable for the adjustment of the slat angle when the venetian blinds are fully drawn ($H_{ismaxX}=1$) on façade number X, at time step k+i
$s_{shX.ph}^{k}$	[-]	Vector ($N_{p}\times \;1$) of the control variable for the adjustment of the slat angle when the venetian blinds are fully drawn ($H_{ismaxX}=1$) on façade number X, over the prediction horizon starting at time step k
$T_{ADI}^{k+i}$	[°C]	Temperature of internal mass (e.g., furniture) in the room, at time step k+i
$T_{Air}^{k+i}$	[°C]	Temperature of the air in the room (thermal zone), at time step k+i
$T_{airSupply}^{k+i}$	[°C]	Temperature of the supply air from the mechanical ventilation system, at time step k+i
$T_{Amb}^{k+i}$	[°C]	Ambient air temperature, at time step k+i
$T_{COMFORT;MAX}$	[°C]	Upper bound of the room air temperature, for thermal comfort
$T_{COMFORT;MIN}$	[°C]	Lower bound of the room air temperature, for thermal comfort
$T_{NB}^{k+i}$	[°C]	Average air temperature of the neighbouring rooms, weighted with the surface area of the common walls, at time step k+i
$T_{TABS}$	[°C]	Temperature of the Thermally Activated Building Structure (TABS): heating/cooling slab
$u_{k+i}$	[-]	Vector ($(1+2+3\cdot N_{F})\times \;1$) of control variables, at time step k+i
$U_{ext}$	[W/K]	Heat transfer coefficient, reversed value of the equivalent thermal resistance through all the façades, from ambient air $T_{Amb}$ to room air $T_{Air}$, identified parameter of the grey-box model
$U_{int}$	[W/K]	Heat transfer coefficient, reversed value of the equivalent thermal resistance through all internal walls, from the average neighbouring room air temperature $T_{NB}$ to the room air temperature $T_{Air}$, identified parameter of the grey-box model
$U_{k}$	[-]	Vector ($N_{u}\;N_{p}\times \;1$) of the predicted profile of the control variables, starting at time step k
${\dot{V}}_{vent}^{k+i}$	[m³/s]	Ventilation air flow at time step k+i
$x_{k+i}$	[°C]	Vector ($N_{x}\times \;1$) of state variables (temperature nodes) at time step k+i
X	[-]	Façade orientation index
$y_{k+i}$	[°C]	Vector (1×1) of output variable at time step k+i
$Y_{k+1}\;$	[°C]	Vector ($N_{p}\times \;1$) of the predicted profile of the output variable, at time step k
		
α	[°]	Sun position: elevation, parameter of the façade model characteristic map
γ	[°]	Slat angle of the external venetian blinds, parameter of the façade model characteristic map
$\gamma_{max}$	[°]	Maximum slat angle of the external venetian blinds (technical limitation, fully closed)
$\gamma_{limit}^{k+i}$	[°]	Limit slat angle of the external venetian blinds to block the direct solar irradiance at time step k+i
Δt	[s]	Time step duration
$\theta_{X}^{k+i}$	[°]	Incidence angle of direct solar radiation on façade X at time step k+i
κ	[-]	Transmission exponent, depending on the glazing type
$\rho_{air}$	[kg/m³]	Ventilation air density
$\tau_{sh.B}$	[-]	Transparency of the shading system (direct solar irradiance)
$\tau_{sh.D.limit}^{k+i}$	[-]	Transparency of the shading system (diffuse solar irradiance) for the limit slat angle $\gamma_{limit}^{k+i}$ at time step k+i
$\tau_{sh.D.min}$	[-]	Transparency of the shading system (diffuse solar irradiance) for the maximum slat angle $\gamma_{max}$
ϕ	[°]	Sun position: azimuth, parameter of the façade model characteristic map
$\phi_{A}$	[-]	Correction factor of incoming solar irradiance, identified parameter of the grey-box model
$\Phi_{k}$	[°C]	Matrix ($N_{p}\times N_{u}N_{p}$) of the linear terms relative to the control variables $U_{k}$, over the prediction horizon starting at time step k

### 2 Data-driven Model Predictive Control approach

#### 2.1 System architecture

The proposed DMPC strategy aims to optimize building operation by maximizing demand-side flexibility. The system is designed to process information on both energy supply, such as market price forecasts for electrical energy, and energy demand, including thermal zone forecasts. At its core, the strategy employs a predictive model, here a grey-box state-space model, capable of forecasting energy consumption in thermal zones while considering comfort constraints over the prediction horizon. A key focus of the algorithm is solar load identification, particularly important for buildings with glass façade elements, enabling effective management of solar gains. By integrating real-time data collection with numerical optimization methods for parameter estimation and control trajectory calculation, the DMPC system makes informed decisions to enhance building performance, reduce energy costs, and improve sustainability. This comprehensive approach ensures that the system can effectively balance energy consumption and occupant comfort, adapting to varying conditions and maximizing the use of available resources.

**Fig. 1:**
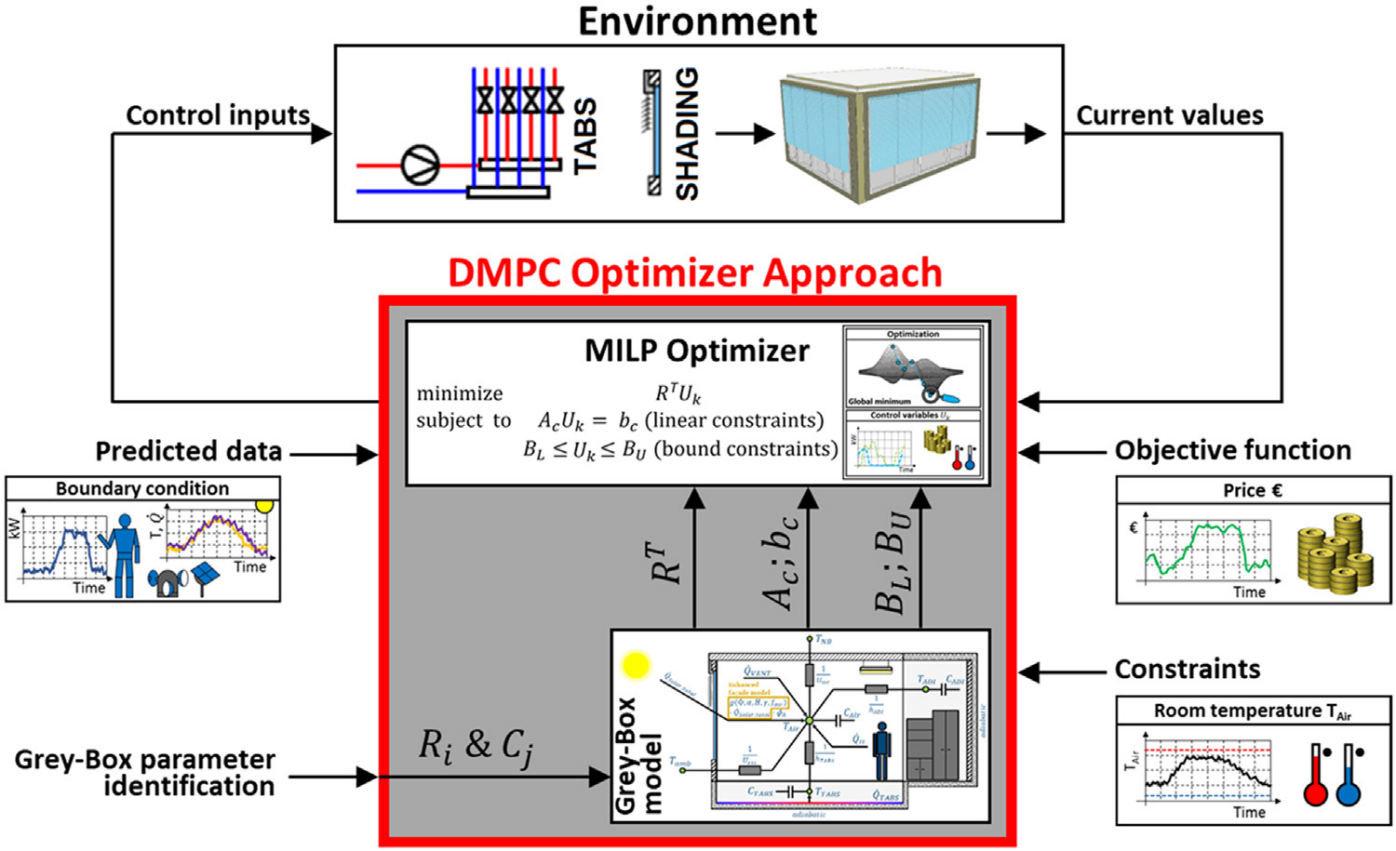
Overview of the Data-driven Model Predictive Control (DMPC) architecture.

Fig. 1 illustrates the structure of the control strategy. Based on the state of the thermal zone and the environment (forecast of the weather and of internal loads), the objective function is calculated, taking into account comfort criteria as constraints on the room air temperature (thermal comfort). Several optimization scenarios can be considered: minimizing either energy costs (€), energy demand (kWh) or CO_2_ emissions (kg_CO2eq_). The optimization algorithm finds the most effective profile of the control variables over the control horizon. The control strategy includes profiles for external venetian blinds (height and slat angle) and for cooling and heating surfaces.

The core of this article is the presentation of the detailed formulation of the proposed grey-box model for DMPC application.

#### 2.2 Required matrix representation for the Mixed Integer Linear Programming solver

The developed DMPC algorithm relies on the Mixed Integer Linear Programming (MILP) solver-based optimization, which requires the translation of the problem into matrix form. One advantage of this approach is the generally faster solving times. MILP can handle a wide range of real-world problems, providing reliably solutions even under complex constraints [31]. It is therefore well suited for energy optimisation problems in buildings, which include discrete and continuous variables, as well as multiple design constraints. The MILP solver from GUROBI® is used [32]. It solves problems of the form:

(1) $$\left\{ \begin{array}{cc} \text{minimize} & {U_{k}}^{⊤}QU_{k}\;+\;R^{⊤}x\;+\alpha \\ \text{subject}\;\text{to} & A_{c}U_{k}=\;b_{c}\;\left(\text{linear}\;\text{constraints}\right)\\ \; & B_{L}\leq U_{k}\leq B_{U}\;\left(\text{bound}\;\text{constraints}\right)\\ \; & \text{some}\;Uj\;\text{integral}\;\left(\text{integrality}\;\text{constraints}\right)\\ \; & {U_{k}}^{⊤}Q_{c}U_{k}\;+\;q^{⊤}U_{k}\leq \beta \;\left(\text{quadratic}\;\text{constraints}\right)\\ \; & \text{some}\;U_{i}\;\text{in}\;\text{SOS}\;\left(\text{special}\;\text{ordered}\;\text{set}\;\text{constraints}\right)\\ \; & \text{min},\text{max},\text{abs},\text{or},\ldots \;\left(\text{general}\;\text{constraints}\right) \end{array}\right.$$

For each optimization run, the following information is required:

• Prediction of the upcoming weather and internal loads: in a simulation study, measurement data can be used as ideal forecasts
• Forecast of the electrical energy prices, from the grid operator
• Historical data for parameter identification of the grey box model

### 3 Formulation of the grey-box model

#### 3.1 Overview of the proposed grey-box model

**Fig. 2:**
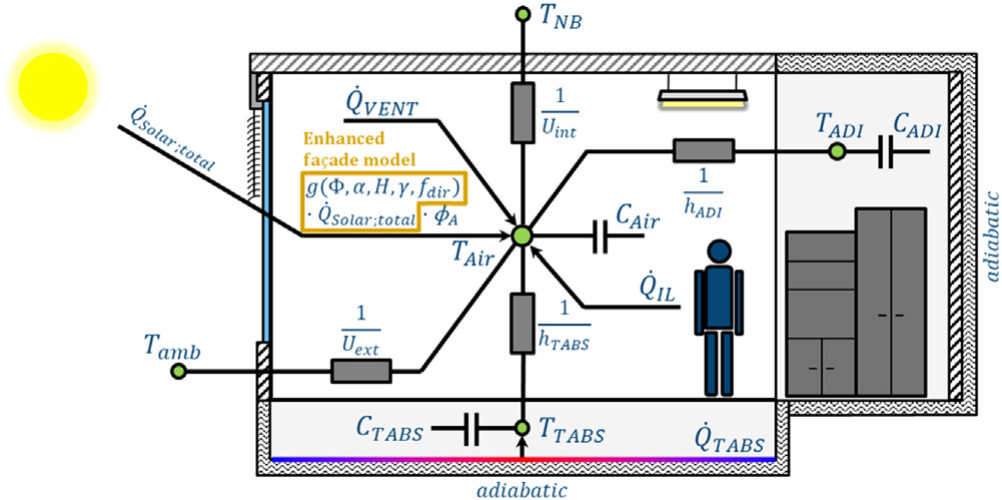
Adapted Grey-Box model (4R3C-network) to model the thermal zone (adapted from [27]).

The DMPC algorithm employs a grey-box model, specifically a 4R3C network (as illustrated in Fig. 2), to represent the thermal zone using a simplified resistance and capacitance (RC) network. With a limited number of parameters, this state-space model is well-suited for data-driven parameter identification.

Compared to the 3R3C network introduced in [27], the 4R3C network includes an additional resistance to model heat transfer through internal walls to or from neighbouring rooms, at temperature $T_{NB}$. This temperature $T_{NB}$ is calculated as the area-weighted average temperature, taking into account the walls in common with the neighbouring rooms.

The selected model structure incorporates a single resistance and capacitance for the heating and cooling elements, as heating and cooling do not occur simultaneously or on the same day. Preliminary tests on a reference building showed that modelling separately heating and cooling does not improve significantly the model accuracy. In many buildings, a single TABS is actually used for heating and cooling.

Modelling the solar gains accurately is critical. Therefore, a specific façade model enhances the performance of the grey-box model. The façade model is represented by a characteristic map which has been established through simulations with a white-box model of a façade, calibrated on measurements from a reference building. More details about the development of this façade model can be found in [33], where measurement data are analysed in details, and in [28], where the characteristic map is presented. In essence, the overall Solar Heat Gain Coefficient $g(\phi ,\alpha ,\;H,\gamma ,f_{dir})$ through the façade is set as a function of the sun position –azimuth angle ϕ (°) and elevation angle α (°)–, the share of shaded glazed façade in height H (0-1), with the slat angle γ (°), and the share $f_{dir}$ (0-1) of direct solar radiation in the global solar radiation ${\dot{Q}}_{Solar;total}$ (W) in the façade plane. The solar gain ${\dot{Q}}_{Solar;in}$ (W) is then corrected by a factor $\phi_{A}\;$(-), which is an identified parameter:

(2) $${\dot{Q}}_{Solar;in}=\phi_{A}\cdot g\left(\phi ,\alpha ,\;H,\gamma ,f_{dir}\right)\cdot {\dot{Q}}_{Solar;total}$$

In buildings with a double flow mechanical ventilation system, the ventilation heat flow ${\dot{Q}}_{VENT}$ can be calculated by balancing the heat flows from the supply air at temperature $T_{airSupply}$ (°C) and from the exhaust air at temperature $T_{Air}$ (°C): ${\dot{Q}}_{VENT}={\dot{V}}_{vent}\cdot \rho_{air}\cdot c_{p;air}\cdot \left(T_{airSupply}-T_{Air}\right)$, knowing the ventilation air flow rate ${\dot{V}}_{vent}$ (m³/s) and the air properties: $\rho_{air}$ (kg/m³) the density and $c_{p;air}$ (J/(kg.K)) the thermal capacity.

Other internal loads are taken into account in ${\dot{Q}}_{IL}$ (W), including for example heat gains from power consumption and occupants.

The 4R3C network, shown in Fig. 2, considers three states: the air temperature state ("Air"), the thermally activated building structure temperature state ("TABS"), and the adiabatic wall and furniture temperature state ("ADI"). This network can be described by the following differential equations:

(3) $$\frac{dT_{TABS}}{dt}=\frac{1}{C_{TABS}}\cdot \left({\dot{Q}}_{TABS}+h_{TABS}\cdot \left(T_{Air}-T_{TABS}\right)\right)$$

(4) $$\frac{dT_{ADI}}{dt}=\frac{1}{C_{ADI}}\cdot \left(h_{ADI}\cdot \left(T_{Air}-T_{ADI}\right)\right)$$

(5) $$\begin{array}{ccc} \frac{dT_{Air}}{dt} & = & \frac{1}{C_{Air}}\cdot [\phi_{A}\cdot g\left(\phi ,\alpha ,H,\gamma ,f_{dir}\right)\cdot {\dot{Q}}_{Solar;total}\\ & & +\;{\dot{V}}_{vent}\cdot \rho_{air}\cdot c_{p;air}\cdot \left(T_{airSupply}-T_{Air}\right)\;+{\dot{Q}}_{IL}+h_{TABS}\cdot \left(T_{TABS}-T_{Air}\right)\\ & & +\;h_{ADI}\cdot \left(T_{ADI}-T_{Air}\right)\;+\;U_{ext}\cdot \left(T_{amb}-T_{Air}\right)\;+U_{int}\cdot \left(T_{NB}-T_{Air}\right)] \end{array}$$

In these equations, the terms are of different types. Eight terms represent the parameters of the 4R3C model: $C_{TABS},h_{TABS},C_{ADI},h_{ADI},C_{Air}\Phi_{A},U_{ext},U_{int}$. Eight further terms denote the boundary conditions: ${\dot{Q}}_{TABS},g\left(\Phi ,\alpha ,H,\gamma ,f_{dir}\right),{\dot{Q}}_{Solar;total},{\dot{V}}_{vent},T_{airSupply},{\dot{Q}}_{IL},T_{amb},T_{NB}$. Three terms indicate the state variables: $T_{Air}$, $T_{ADI}$ and $T_{TABS}$. In contrast to Klanatsky et al. [27], it is recommended to include the three initial state temperatures in the parameters to be identified, in addition to the 8 parameters (4R3C) or 7 parameters (3R3C) of the RC model. A total of 11 parameters (4R3C) or 10 parameters (3R3C) must therefore be determined. The parameter identification task (e.g. minimizing the sum of squared errors) can be performed on the basis of past measurement data, such as the dataset published in [34], referring to the grey-box model presentation in [27]. According to the experience on this model, relying on two weeks of data is sufficient for a precise identification: see the parameter study of the grey-box model in [27].

For further details about the chosen model structure and the data-driven parameter identification, readers are kindly referred to the mentioned previous work of Klanatsky et al. [27]. Applications of this grey-box model in a DMPC can be found in the related research article presenting a simulation study [29], and in an article reporting the demonstration in a long-term experiment in real building [30].

#### 3.2 Matrix representation of the time-discrete state-space model

For the solver-based optimization approach, a formulation of the time-discrete state space model in matrix form is implemented. For the mathematical background, refer to [35]. At time step k, the model describes the state of the system over the prediction horizon, e.g. 1 day (24 hours), 2 days (48 hours)… The number of time steps $N_{p}$ in the prediction horizon is determined by defining the time step duration: e.g., with Δt=15min, 1 day has $N_{p}=96$ time steps, 2 days have $N_{p}=192$ time steps…

The following three vectors are defined:

• The vector x of state variables is composed of $N_{x}=3$ temperature nodes. As mentioned, the floor heating and ceiling cooling thermal capacity is represented by a single node: TABS.

(6) $$x=\left[\begin{array}{c} T_{TABS}\\ T_{ADI}\\ T_{Air} \end{array}\right],\;\text{at}\;\text{time}\;\text{step}k+i:\;x_{k+i}=\left[\begin{array}{c} T_{TABS}^{k+i}\\ T_{ADI}^{k+i}\\ T_{Air}^{k+i} \end{array}\right]$$

• The vector y of output variables, in our case the variable of interest for the comfort $T_{Air}$:

(7) $$y=\left[T_{Air}\right],\;\text{at}\;\text{time}\;\text{step}k+i:y_{k+i}=\left[T_{Air}^{k+i}\right]$$

• The vector u of control variables contains $N_{u}=1+2+3\cdot N_{F}$ control variables, with $N_{F}$ the number of external façades of the thermal zone. The first variable (first row), always equals to 1, is used to introduce the constant terms of the equations. The two signals for heating and cooling are respectively $CTRL_{FH}$ and $CTRL_{CC}$. Then come the shading control parameters, for each of the $N_{F}$ façades: the height of the shutters $h_{shX}$, a helping variable $H_{ismaxX}$, checking if the maximum shaded height is reached, and the slat angle correction $s_{shX}$. This last parameter adjusts the share of solar gains from diffuse radiations passing through the shutters, linearly between $s_{shX}=1$ giving the maximum solar gains for the slat angle limit $\gamma_{limit}$ (minimum slat angle which blocks direct sun rays) and $s_{shX}=0$ giving the minimum solar gains for the maximum slat angle $\gamma_{max}$ (here 87°). Computing $s_{shX}$ depending on the maximum shaded height $H_{shX}$ is managed by constraints, as explained in section 5.2.2.

(8) $$u=\left[\begin{array}{c} 1\\ CTRL_{FH}\\ CTRL_{CC}\\ h_{sh1}\\ H_{ismax1}\\ s_{sh1}\\ h_{sh2}\\ \ldots \end{array}\right],\;\text{at}\;\text{time}\;\text{step}\;k+i:\;u_{k+i}=\left[\begin{array}{c} 1\\ CTRL_{FH}^{k+i}\\ CTRL_{CC}^{k+i}\\ h_{sh1}^{k+i}\\ H_{ismax1}^{k+i}\\ s_{sh1}^{k+i}\\ h_{sh2}^{k+i}\\ \ldots \end{array}\right]$$

The thermal load to the TABS ${\dot{Q}}_{TABS}$ is then defined depending on the season, for each time step k+i, as: ${\dot{Q}}_{FH}^{k+i}$ for heating and ${\dot{Q}}_{CC}^{k+i}$ for cooling, based on the nominal (maximal) load for heating ${\dot{Q}}_{FH_{n}}^{k+i}$ and for cooling ${\dot{Q}}_{CC_{n}}^{k+i}$:

(9) $$\left\{ \begin{array}{c} {\dot{Q}}_{FH}^{k+i}={\dot{Q}}_{FH_{n}}^{k+i}CTRL_{FH}^{k+i}\\ {\dot{Q}}_{CC}^{k+i}={\dot{Q}}_{CC_{n}}^{k+i}CTRL_{CC}^{k+i} \end{array}\right.$$

The nominal load for heating ${\dot{Q}}_{FH_{n}}^{k+i}$ and for cooling ${\dot{Q}}_{CC_{n}}^{k+i}$ is noted time dependent, but fixed values can be used, which can be noted: ${\dot{Q}}_{FH_{n}}$ and ${\dot{Q}}_{CC_{n}}$.

For each predicted time step k+i the state space model can then be written, in time discrete form, as follow:

(10) $$\left\{ \begin{array}{c} x_{k+i+1}=A_{k+i}\cdot x_{k+i}+B_{k+i}\cdot u_{k+i}\\ y_{k+i}=C\cdot x_{k+i} \end{array}\right.$$

with $A_{k+i}$, $B_{k+i}$ and C the matrices containing the coefficients corresponding to the differential equations. Each row corresponds to an equation, each column corresponds to a variable:

• $A_{k+i}$ contains the linear terms relative to the state variables $x_{k+i}$ (temperatures):

(11) $$A_{k+i}=\left[\begin{array}{ccc} 1-\frac{h_{TABS}\Delta t}{C_{TABS}} & 0 & \frac{h_{TABS}\Delta t}{C_{TABS}}\\ 0 & 1-\frac{h_{ADI}\Delta t}{C_{ADI}} & \frac{h_{ADI}\Delta t}{C_{ADI}}\\ \frac{h_{TABS}\Delta t}{C_{Air}} & \frac{h_{ADI}\Delta t}{C_{Air}} & 1-\left(U_{ext}+U_{int}+h_{TABS}+h_{ADI}+{\dot{V}}_{vent}^{k+i}\rho_{air}c_{p;air}\right)\frac{\Delta t}{C_{Air}} \end{array}\right]$$

when considering the 3R3C-network, the value of $U_{int}$ is constant, equal to 0.

• $B_{k+i}$ contains the linear terms relative to the control variables $u_{k+i}$:

(12) $$B_{k+i}=\left[\begin{array}{cccccccccc} 0 & \frac{{\dot{Q}}_{FH_{n}}^{k+i}\Delta t}{C_{TABS}} & \frac{{\dot{Q}}_{CC_{n}}^{k+i}\Delta t}{C_{TABS}} & 0 & 0 & 0 & 0 & 0 & 0 & 0\\ 0 & 0 & 0 & 0 & 0 & 0 & 0 & 0 & 0 & 0\\ B_{3,1}^{k+i} & 0 & 0 & B_{3,4}^{k+i} & 0 & B_{3,6}^{k+i} & B_{3,7}^{k+i} & 0 & B_{3,9}^{k+i} & \ldots \end{array}\right]$$

with:

$$B_{3,1}^{k+i}=\frac{\Delta t}{C_{Air}}\left({\dot{Q}}_{IL}^{k+i}+\phi_{A}{\dot{Q}}_{sol.in.noSh}^{k+i}+U_{ext}\;T_{Amb}^{k+i}+U_{int}\;T_{NB}^{k+i}+{\dot{V}}_{vent}^{k+i}\rho_{air}c_{p;air}T_{airSupply}^{k+i}\right)$$

$$B_{3,4}^{k+i}=\frac{\Delta t}{C_{Air}}\phi_{A}\;g_{win}\;A_{1}\;\left({\dot{q}}_{sol.G.cor.Sh.limit.1}^{k+i}-{\dot{q}}_{sol.G.cor.1}^{k+i}\right)$$

$$B_{3,6}^{k+i}=\frac{\Delta t}{C_{Air}}\phi_{A}\;g_{win}\;A_{1}\left(\left(\tau_{sh.D.min}-\tau_{sh.D.limit}^{k+i}\right)\;r_{D}\;{\dot{q}}_{sol.D.1}^{k+i}\right)$$

$$B_{3,7}^{k+i}=\frac{\Delta t}{C_{Air}}\phi_{A}\;g_{win}\;A_{2}\left({\dot{q}}_{sol.G.cor.Sh.limit.2}^{k+i}-{\dot{q}}_{sol.G.cor.2}^{k+i}\right)$$

$$B_{3,9}^{k+i}=\frac{\Delta t}{C_{Air}}\phi_{A}\;g_{win}\;A_{2}\left(\left(\tau_{sh.D.min}-\tau_{sh.D.limit}^{k+i}\right)\;r_{D}\;{\dot{q}}_{sol.D.2}^{k+i}\right)$$

$$B_{3,1+3\cdot X}^{k+i}=\frac{\Delta t}{C_{Air}}\phi_{A}\;g_{win}\;A_{X}\left({\dot{q}}_{sol.G.cor.Sh.limit.X}^{k+i}-{\dot{q}}_{sol.G.cor.X}^{k+i}\right)$$

(13) $$B_{3,3+3\cdot X}^{k+i}=\frac{\Delta t}{C_{Air}}\phi_{A}\;g_{win}\;A_{X}\left(\left(\tau_{sh.D.min}-\tau_{sh.D.limit}^{k+1}\right)\;r_{D}\;{\dot{q}}_{sol.D.X}^{k+i}\right)$$

$B_{3,1}^{k+i}$ contains the terms that are independent from the control strategy. This includes a term for the ventilation heat flow (${\dot{V}}_{vent}^{k+i}\rho_{air}c_{p;air}T_{airSupply}^{k+i}$) and a term for other internal loads (${\dot{Q}}_{IL}^{k+i}$). Here too, when considering the 3R3C-network, $U_{int}$ is equal to 0. The term $\phi_{A}{\dot{Q}}_{sol.in.noSh}^{k+i}$ represents the solar gains without any shading, summing the solar gains of each façade X, from the set of façades F:

(14) $${\dot{Q}}_{sol.in.noSh}^{k+i}=\sum_{X\in F}g_{win}\;A_{X}\;{\dot{q}}_{sol.G.cor.X}^{k+i}$$

with:

$g_{win}$ (-) the overall solar heat gain coefficient, which is a characteristic of the glass façade, $A_{X}$ (m²) the surface area of façade X, ${\dot{q}}_{sol.G.cor.X}^{k+i}$ (W/m²) the specific global solar radiation in the plane of façade X, corrected for the different transmittance of direct and diffuse solar radiations through a glazing, defined by:

(15) $${\dot{q}}_{sol.G.cor.X}^{k+i}=r_{B.X}^{k+i}\;{\dot{q}}_{sol.B.X}^{k+i}+r_{D}\;{\dot{q}}_{sol.D.X}^{k+i}$$

with:

${\dot{q}}_{sol.B.X}^{k+i}$the specific direct solar radiation (${\;}_{B}$ for beam) in the plane of façade X and ${\dot{q}}_{sol.D.X}^{k+i}$ the specific diffuse solar radiation in the plane of façade X, which is calculated as the difference between global solar radiation ${\dot{q}}_{sol.G.X}^{k+i}$ and direct solar radiation ${\dot{q}}_{sol.B.X}^{k+i}$, both in the plane of façade X:

(16) $${\dot{q}}_{sol.D.X}^{k+i}={\dot{q}}_{sol.G.X}^{k+i}-{\dot{q}}_{sol.B.X}^{k+i}$$

$r_{B.X}^{k+i}$ and $r_{D}$ correction coefficients, accounting for the different transmittance of direct and diffuse solar radiation through a glazing. These coefficients are defined based on the Austrian standard H 6040 (see e.g. equation 198, section 11.4, page 86) [36]:$r_{B.X}^{k+i}=1-{\left(1-\cos\theta_{X}^{k+i}\right)}^{\kappa }$ for direct radiation, depending on the incidence angle $\theta_{X}^{k+i}$ (°) of direct solar radiation on façade X, and on the transmission exponent κ, depending on the glazing type –for 3-pane absorption solar control glass: κ=1.5 $r_{D}=\frac{\kappa \;(\kappa +3)}{\left(\kappa +1)(\kappa +2\right)}$ for diffuse radiation, depending solely on the same transmission exponent κ.

The following $B_{3,X}^{k+i}$ terms represent the influence of the shading settings on the solar gains: $B_{3,4}^{k+i}$ and $B_{3,6}^{k+i}$ correspond to façade X=1, $B_{3,4}^{k+i}$ defines the height control and $B_{3,6}^{k+i}$ the slat angle control. Similarly, $B_{3,7}^{k+i}$ and $B_{3,9}^{k+i}$ correspond to façade X=2, and $B_{3,1+3\cdot X}^{k+i}$ and $B_{3,3+3\cdot X}^{k+i}$ represent façade number X.

The term $B_{3,1+3\cdot X}^{k+i}$ is multiplied by the height control signal $h_{shX}^{k+i}$, giving the reduction of solar gains achieved by the part of the glazing that is shaded. The solar radiation passing through the venetian blinds is calculated for the limit slat angle $\gamma_{limit}^{k+i}$, which is the minimum angle blocking direct sun light at a certain time step k+i, is calculated. This $B_{3,1+3\cdot X}^{k+i}$ term is based on:

(17) $${\dot{q}}_{sol.G.cor.Sh.limit.X}^{k+i}=\tau_{sh.B}\;r_{B.X}^{k+i}\;{\dot{q}}_{sol.B.X}^{k+i}+\tau_{sh.D.limit}^{k+i}\;r_{D}\;{\dot{q}}_{sol.D.X}^{k+i}$$

with:

$\tau_{sh.B}$ the remaining transmittance of direct sun light through the venetian blinds, when the slats block the direct sun rays: a value of $\tau_{sh.B}=0.03$ can be recommended based on the mentioned façade model.

$\tau_{sh.D.limit}^{k+i}$ the transmittance of diffuse sun light through the venetian blinds depending on the limit slat angle $\gamma_{limit}^{k+i}$. Extracting information from the charactersistic map of the developed façade model, $\tau_{sh.D.limit}^{k+i}$ can be defined by a polynomial equation of fourth order:

(18) $$\tau_{sh.D.limit}^{k+i}=2.906146\cdot 10^{-9}\gamma {{}_{limit}^{k+i}}^{4}-9.641525\cdot 10^{-9}\gamma {{}_{limit}^{k+i}}^{3}-6.901166\cdot 10^{-5}\gamma {{}_{limit}^{k+i}}^{2}+3.276592\cdot 10^{-5}\gamma_{limit}^{k+i}+0.360238$$

The term $B_{3,3+3\cdot X}^{k+i}$ is multiplied by the slat angle correction signal $s_{shX}^{k+i}$, adjusting the reduction of solar gains caused by the slat angle variation from its current limit value $\gamma_{limit}^{k+i}$ to its maximal value $\gamma_{max}$. This term assumes a linear dependence of the diffuse transmittance between these slat angle values, varying from $\tau_{sh.D.limit}^{k+i}$ at $\gamma_{limit}^{k+i}$ to $\tau_{sh.D.min}$ at $\gamma_{max}$. According to the façade model, the fully tilted position corresponds to the maximum angle technically possible of $\gamma_{max}=87^{\circ }$. The minimum diffuse transmittance can be set to $\tau_{sh.D.min}=0.07$.

In this implementation, the façade model can be summed up by only 9 parameters:

о 4 fixed parameters: $\tau_{sh.B}=0.03$, $\tau_{sh.D.min}=0.07$, κ=1.5, used to define $r_{D}$ and $r_{B.X}^{k+i}$ according to the austrian standard H 6040 [36], and $g_{win}=0.5$. Note that the parameter $g_{win}$ should be known from the manufacturer of the glazing, so that the parameter $\phi_{A}$ is expected to be close to 1. If unknown, $g_{win}$ can also be identified together with parameter $\phi_{A}$.
о 5 parameters of the polynomial equation, defining $\tau_{sh.D.limit}^{k+i}$ (see Eq. (18)).

This reasonable level of complexity keeps the reproducibility of this façade model accessible, considering that other venetian blinds might behave similarly.

• C extracts the output variable $y_{k+i}$ (corresponding to $T_{Air}$) from the state variable vector $x_{k+i}$:

(19) $$C=\left[\begin{array}{ccc} 0 & 0 & 1 \end{array}\right]$$

To predict the air temperature in the thermal zone over the prediction horizon and solve the optimization problem (hourly or daily), the output variable at each predicted time step $y_{k+i}$ needs to be expressed as a function of the present values of temperatures $x_{k}$ and the control variables over the prediction horizon $u_{k}$,$u_{k+1}$,$u_{k+2}$,…$u_{k+i}$:

(20) $$\begin{array}{ccc} y_{k+1} & = & C\cdot x_{k+1}\\ & = & C\cdot A_{k}\cdot x_{k}+C\cdot B_{k}\cdot u_{k}\\ y_{k+2} & = & C\cdot A_{k+1}\cdot x_{k+1}+C\cdot B_{k+1}\cdot u_{k+1}\\ & & C\cdot A_{k+1}\cdot A_{k}\cdot x_{k}+C\cdot A_{k+1}\cdot B_{k}\cdot u_{k}+C\cdot B_{k+1}\cdot u_{k+1}\\ \ldots & & \\ y_{k+N_{p}} & \end{array}$$

We can note $Y_{k+1}=\left[\begin{array}{c} y_{k+1}\\ y_{k+2}\\ \vdots \\ y_{k+N_{p}} \end{array}\right]$ the predicted profile of output variables $y_{k+i}$ from time step k+1 to $k+N_{p}$ and

$U_{k}=\left[\begin{array}{c} u_{k}\\ u_{k+1}\\ \vdots \\ u_{k+N_{p}-1} \end{array}\right]$ the predicted profile of control variables $u_{k+i}$ from time step k to time step $k+N_{p}-1$. Mind that $Y_{k+1}$ is $N_{p}\times \;1$ vector, while $x_{k}$ is $N_{u}\times \;1$ vector and $U_{k}$ is $N_{u}\;N_{p}\times \;1$ vector. The definition of the$\;y_{k+i}$ terms can then be written in matrix form:

(21) $$Y_{k+1}=F_{k}\cdot x_{k}+\Phi_{k}\cdot U_{k}$$

with $F_{k}$ an $N_{p}\times N_{x}$ matrix:

(22) $$F_{k}=\left[\begin{array}{c} CA_{k}\\ CA_{k+1}A_{k}\\ \vdots \\ CA_{k+N_{p}-1}A_{k+N_{p}-2}..A_{k+1}A_{k} \end{array}\right]$$

and $\Phi_{k}$ an $N_{p}\times N_{u}N_{p}$ matrix:

(23) $$\Phi_{k}=\left[\begin{array}{ccccc} CB_{k} & 0 & 0 & \cdots & 0\\ CA_{k+1}B_{k} & CB_{k+1} & 0 & \cdots & 0\\ CA_{k+2}A_{k+1}B_{k} & CA_{k+2}B_{k+1} & CB_{k+2} & \ddots & \vdots \\ \vdots & \vdots & \ddots & \ddots & 0\\ CA_{k+N_{p}-1}A_{k+N_{p}-2}..A_{k+1}B_{k} & CA_{k+N_{p}-1}..A_{k+2}B_{k+1} & \cdots & CA_{k+N_{p}-1}B_{k+N_{p}-2} & CB_{k+N_{p}-1} \end{array}\right]$$

### 4 Formulation of the objective function

The objective function can express several variables integrated over the entire prediction horizon. To emphasise how easy it is to modify the target variable of the objective function, three examples are presented here: objective functions minimizing energy costs, energy demand or CO_2_ emissions.

The costs $J_{cost}$, the energy demand $J_{energy}$ and the CO_2_ emissions $J_{co2}$, for heating and cooling over the entire prediction horizon, can be expressed in matrix form as follow:

(24) $$J_{cost}=R_{cost}^{T}\cdot U_{k}\;J_{energy}=R_{energy}^{T}\cdot U_{k}\;J_{co2}=R_{co2}^{T}\cdot U_{k}$$

where $R_{cost}$, $R_{energy}$ and $R_{co2}$ are $N_{u}N_{p}\times \;1$ column vectors:

(25) $$R_{cost}=\left[\begin{array}{c} \begin{array}{c} 0\\ \frac{{\dot{Q}}_{FH_{n}}^{k}}{COP_{FH}}\Delta t\cdot EP^{k}\\ \frac{{\dot{Q}}_{CC_{n}}^{k}}{EER_{CC}}\Delta t\cdot EP^{k} \end{array}\\ 0\\ 0\\ 0\\ \vdots \\ 0\\ \frac{{\dot{Q}}_{FH_{n}}^{k+i}}{COP_{FH}}\Delta t\cdot EP^{k+i}\\ \frac{{\dot{Q}}_{CC_{n}}^{k+i}}{EER_{CC}}\Delta t\cdot EP^{k+i}\\ 0\\ 0\\ 0\\ \vdots \\ 0\\ \frac{{\dot{Q}}_{FH_{n}}^{k+N_{p}-1}}{COP_{FH}}\Delta t\cdot EP^{k+N_{p}-1}\\ \frac{{\dot{Q}}_{CC_{n}}^{k+N_{p}-1}}{EER_{CC}}\Delta t\cdot EP^{k+N_{p}-1}\\ 0\\ 0\\ 0 \end{array}\right],\;R_{energy}=\left[\begin{array}{c} \begin{array}{c} 0\\ \frac{{\dot{Q}}_{FH_{n}}^{k}}{COP_{FH}}\Delta t\\ \frac{{\dot{Q}}_{CC_{n}}^{k}}{EER_{CC}}\Delta t \end{array}\\ 0\\ 0\\ 0\\ \vdots \\ 0\\ \frac{{\dot{Q}}_{FH_{n}}^{k+i}}{COP_{FH}}\Delta t\\ \frac{{\dot{Q}}_{CC_{n}}^{k+i}}{EER_{CC}}\Delta t\\ 0\\ 0\\ 0\\ \vdots \\ 0\\ \frac{{\dot{Q}}_{FH_{n}}^{k+N_{p}-1}}{COP_{FH}}\Delta t\\ \frac{{\dot{Q}}_{CC_{n}}^{k+N_{p}-1}}{EER_{CC}}\Delta t\\ 0\\ 0\\ 0 \end{array}\right],R_{co2}=\left[\begin{array}{c} \begin{array}{c} 0\\ \frac{{\dot{Q}}_{FH_{n}}^{k}}{COP_{FH}}\Delta t\cdot CF^{k}\\ \frac{{\dot{Q}}_{CC_{n}}^{k}}{EER_{CC}}\Delta t\cdot CF^{k} \end{array}\\ 0\\ 0\\ 0\\ \vdots \\ 0\\ \frac{{\dot{Q}}_{FH_{n}}^{k+i}}{COP_{FH}}\Delta t\cdot CF^{k+i}\\ \frac{{\dot{Q}}_{CC_{n}}^{k+i}}{EER_{CC}}\Delta t\cdot CF^{k+i}\\ 0\\ 0\\ 0\\ \vdots \\ 0\\ \frac{{\dot{Q}}_{FH_{n}}^{k+N_{p}-1}}{COP_{FH}}\Delta t\cdot CF^{k+N_{p}-1}\\ \frac{{\dot{Q}}_{CC_{n}}^{k+N_{p}-1}}{EER_{CC}}\Delta t\cdot CF^{k+N_{p}-1}\\ 0\\ 0\\ 0 \end{array}\right]$$

with $EP^{k+i}$ (€/J) the energy price profile, at times step k+i, and $CF^{k+i}$ (kgCO_2_e/J) the conversion factors from energy in J to equivalent CO_2_ emissions in kgCO_2_e, at times step k+i. $COP_{FH}$ (-) is the average Coefficient Of Performance of the heat pump in the heating case. $EER_{CC}$ (-) is the average Energy Efficiency Ratio of the heat pump in the cooling case.

### 5 Formulation of the constraint functions

#### 5.1 Constraints on the control variables

The control variables can be of two types: continuous variables that vary freely between 0 and 1 (continuous type), and discrete variables that take a finite number of values, here 0 and 1 (integer type). To enable operation of the heat pump at one or several intermediate loads, the variable bounds in integer type can be adapted as desired: e.g. {0, 1, 2} can be used to enable operation at 50% of the nominal power. In the proposed implementation, the following settings apply:

• For heating and cooling, the lower bound of $CTRL_{FH}$ and $CTRL_{CC}$ is zero and the upper bound is one (nominal load). Two variants of the variable type are suggested for the $CTRL_{FH}$ and $CTRL_{CC}$variables: one with integer type and one with continuous type.
• For setting the shaded height $h_{shX}$ (continuous variable), the minimum is zero, representing the shutters fully open, and the maximum shaded height is one. An additional control variable for the shading is $H_{ismaxX}$ (binary variable), equal to 0 or 1. The slat angle correction $s_{shX}$ is a continuous variable, varying from $s_{shX}=0$ when the slat angle is at its current limit $\gamma_{limit}$, which is the minimum angle for blocking the direct sunlight, and $s_{shX}=1$ when the slat angle is maximum $\gamma_{max}$.

In summary, two variants are proposed, with integer or continuous variables for heating and cooling control:

(26) $$\begin{array}{c} \left\{ \begin{array}{c} \mathrm{CTR}L_{\mathrm{FH}}\\ \mathrm{CTR}L_{\mathrm{CC}}\\ h_{\mathrm{shX}}\\ H_{\mathrm{isma}\mathrm{xX}}\\ s_{\mathrm{shX}} \end{array}\begin{array}{c} \in \left[0;1\right]\;\cdots \;\mathrm{inte}\mathrm{ger}\\ \in \left[0;1\right]\;\cdots \;\mathrm{inte}\mathrm{ger}\\ \in \left[0;1\right]\;\cdots \;\text{continuous}\\ \in \left\{0;1\right\}\;\cdots \;\text{binary}\\ \in \left[0;1\right]\;\cdots \;\text{continuous} \end{array}\right.\;\;\;\;\;\;\;\;\;\;\;\;\mathrm{or}\;\;\;\;\;\;\;\;\;\;\;\;\;\left\{ \begin{array}{c} \mathrm{CTR}L_{\mathrm{FH}}\\ \mathrm{CTR}L_{\mathrm{CC}}\\ h_{\mathrm{shX}}\\ H_{\mathrm{isma}\mathrm{xX}}\\ s_{\mathrm{shX}} \end{array}\right.\begin{array}{c} \in \left[0;1\right]\;\cdots \;\mathrm{cont}\mathrm{inuo}\mathrm{us}\\ \in \left[0;1\right]\;\cdots \;\mathrm{cont}\mathrm{inuo}\mathrm{us}\\ \in \left[0;1\right]\;\cdots \;\text{continuous}\\ \in \left\{0;1\right\}\;\cdots \;\text{binary}\\ \in \left[0;1\right]\;\cdots \;\text{continuous} \end{array} \end{array}$$

Using the notation of the solver for the lower bounds $B_{L}$ and the upper bounds $B_{U}$, we have:

(27) $$B_{L}={\left[0\right]}_{N_{u}N_{p}\times \;1}andB_{U}={\left[1\right]}_{N_{u}N_{p}\times \;1}$$

### 5.2 Linear bound constraints

#### 5.2.1 Accounting for thermal comfort

The constraint to keep the zone temperature in a suitable range for thermal comfort can be expressed linearly as:

(28) $$T_{COMFORT;MIN}<T_{Air}<T_{COMFORT;MAX}$$

with

$T_{Air}$ [°C] temperature of the air temperature node (see Fig. 2)

$T_{COMFORT;MIN}$ [°C] lower bound of the air temperature (linear constraint)

$T_{COMFORT;MAX}$ [°C] upper bound of the air temperature (linear constraint)

$T_{Air}$ is defined for each predicted time step in the vector $Y_{k+1}=F_{k}\cdot x_{k}+\Phi_{k}\cdot U_{k}$. So, the thermal constraint applied to all predicted time steps can be written:

(29) $$T_{COMFORT;MIN}\leq F_{k}\cdot x_{k}+\Phi_{k}\cdot U_{k}\leq T_{COMFORT;MAX}$$

For the solver, bound constraints have to be organized as “smaller-than” inequalities. At time step k:

(30) $$A_{c}^{k}\cdot U_{k}\leq b_{c}^{k}$$

The constraints are therefore written as two inequalities:

(31) $$\left\{ \begin{array}{c} \;\Phi_{k}\cdot U_{k}\leq \;T_{COMFORT;MAX}-F_{k}\cdot x_{k}\\ -\Phi_{k}\cdot U_{k}\leq -T_{COMFORT;MIN}+F_{k}\cdot x_{k} \end{array}\right.$$

These linear bound constraints on the room air temperature are summed up in the matrix $A_{cT}^{k}$, containing the linear coefficients:

(32) $$A_{cT}^{k}=\left[\begin{array}{c} \;\Phi_{k}\\ -\Phi_{k} \end{array}\right]$$

and in the vector $b_{cT}^{k}$ of size $2N_{p}\times \;1$, containing the right-hand side coefficients:

(33) $$b_{cT}^{k}=\left[\begin{array}{c} \;T_{COMFORT;MAX}-F_{k}\cdot x_{k}\\ -T_{COMFORT;MIN}+F_{k}\cdot x_{k} \end{array}\right]$$

with $T_{COMFORT;MIN}=21.5{\;}^{\circ }C$ and $T_{COMFORT;MAX}=25.5{\;}^{\circ }C$ for example.

### 5.2.2 Additional linear bound constraints: related to the control variables

Constraints on the shading control variables are defined to activate the slat angle correction $s_{shX}$, only if the maximum shaded height is reached. This is where the helping variable $H_{ismaxX}$ is used.

This constraint applies for each façade $X\in \{1,2,\ldots ,N_{F}\}$, at each time step k+i of the prediction horizon, from time step k to time step $k+N_{p}-1$. $H_{ismaxX}^{k+i}$ can be defined as a function of $h_{shX}^{k+i}$: it is one, if $h_{shX}^{k+i}=1$ and zero, if $h_{shX}^{k+i}<1$:

(34) $$H_{ismaxX}^{k+i}=floor\left(h_{shX}^{k+i}\right)$$

Because this relationship is non-linear, it has to be linearized and organized as “smaller-than or equal-to” inequalities:

(35) $$\left\{ \begin{array}{c} H_{ismaxX}^{k+i}-h_{shX}^{k+i}\leq 0\;\\ h_{shX}^{k+i}-H_{ismaxX}^{k+i}\leq 1-10^{-3} \end{array}\right.$$

(36) $$\left\{ \begin{array}{c} H_{ismaxX}^{k+i}-h_{shX}^{k+i}\leq 0\;\\ h_{shX}^{k+i}-H_{ismaxX}^{k+i}\leq 1-10^{-3} \end{array}\right.$$

An additional constraint forces the slat angle correction $s_{shX}^{k+i}$ to 0, whenever $H_{ismaxX}^{k+i}$ is 0. It can be written as follow:

(37) $$s_{shX}^{k+i}-H_{ismaxX}^{k+i}\leq 0$$

To include these constraints in Eq. (30) ($A_{c}^{k}\cdot U_{k}\leq b_{c}^{k}$), the three inequalities (Eqs. (35), (36), and (37)) have to be verified for the respective control variables on the $N_{F}$ façades, at all $N_{p}$ time steps of the prediction horizon. Therefore, the matrices $A_{cSh1}^{k}$, $A_{cSh2}^{k}$ and $A_{cSh3}^{k}$ containing the coefficients corresponding to the left hand-side of the inequalities are of size $N_{p}\times N_{u}N_{p}$. Following the order of the control variables in $u_{k}$ (Eq. (8)), this gives:

• for inequality (35): $H_{ismaxX}^{k+i}-h_{shX}^{k+i}$

(38) $$\forall X\in \left\{1,2,\ldots ,N_{F}\right\},\forall i\in \left\{0,1,\ldots ,N_{p}-1\right\},\left\{ \begin{array}{c} A_{cSh1}^{k}\left(i+1,i\cdot N_{u}+4+\left(X-1\right)\cdot 3\right)=-1\;\text{defining}\;\text{the}\;\text{coefficient}\;\text{of}\;h_{shX}^{k+i}\\ A_{cSh1}^{k}\left(i+1,i\cdot N_{u}+5+\left(X-1\right)\cdot 3\right)=+1\;\text{defining}\;\text{the}\;\text{coefficient}\;\text{of}\;H_{ismaxX}^{k+i} \end{array}\right.$$

• for inequality (36): $h_{shX}^{k+i}-H_{ismaxX}^{k+i}$

(39) $$\forall X\in \left\{1,2,\ldots ,N_{F}\right\},\forall i\in \left\{0,1,\ldots ,N_{p}-1\right\},\left\{ \begin{array}{c} A_{cSh2}^{k}\left(i+1,i\cdot N_{u}+4+\left(X-1\right)\cdot 3\right)=+1\;\text{defining}\;\text{the}\;\text{coefficient}\;\text{of}\;h_{shX}^{k+i}\\ A_{cSh2}^{k}\left(i+1,i\cdot N_{u}+5+\left(X-1\right)\cdot 3\right)=-1\;\text{defining}\;\text{the}\;\text{coefficient}\;\text{of}\;H_{ismaxX}^{k+i} \end{array}\right.$$

• for inequality (37): $s_{shX}^{k+i}-H_{ismaxX}^{k+i}$

(40) $$\forall X\in \left\{1,2,\ldots ,N_{F}\right\},\forall i\in \left\{0,1,\ldots ,N_{p}-1\right\},\;\left\{ \begin{array}{c} A_{cSh3}^{k}\left(i+1,\;i\cdot \;N_{u}+5+\left(X-1\right)\cdot 3\right)=-1\;\text{defining}\;\text{the}\;\text{coefficient}\;\text{of}\;H_{ismaxX}^{k+i}\\ A_{cSh3}^{k}\left(i+1,\;i\cdot \;N_{u}+6+\left(X-1\right)\cdot 3\right)=+1\;\text{defining}\;\text{the}\;\text{coefficient}\;\text{of}\;s_{shX}^{k+i} \end{array}\right.$$

The vectors $b_{cSh1}$, $b_{cSh2}$ and $b_{cSh3}$, containing the coefficients of the right-hand side of the inequalities are of size $N_{p}\times \;1$:

• for inequality (35): 0

(41) $$b_{cSh1}={\left[0\right]}_{Np\times 1}$$

• for inequality (36): $1-10^{-3}$

(42) $$b_{cSh2}={\left[1-10^{-3}\right]}_{Np\times 1}$$

• for inequality (37): 0

(43) $$b_{cSh3}={\left[0\right]}_{Np\times 1}$$

### 5.2.3 All linear bound constraints

Considering all bound constraints, the matrix $A_{c}^{k}$ of size $5N_{p}\times N_{u}N_{p}$ for the linear coefficients looks like:

(44) $$A_{c}^{k}=\left[\begin{array}{c} A_{cT}^{k}\\ A_{cSh1}^{k}\\ A_{cSh2}^{k}\\ A_{cSh3}^{k} \end{array}\right]$$

and the $b_{c}^{k}$ vector of size $5N_{p}\times \;1$ for the right-hand side coefficients:

(45) $$b_{c}^{k}=\left[\begin{array}{c} b_{cT}^{k}\\ b_{cSh1}\\ b_{cSh2}\\ b_{cSh3} \end{array}\right]$$

### 6 Mixed Integer Linear Programming solver implementation

Based on the Mixed Integer Linear Programming (MILP) solver of GUROBI® [46], the following form of the optimization problem can be solved:

(46) $$\left\{ \begin{array}{ccc} \text{minimize} & \; & R_{cost}^{⊤}U_{k}\;\mathrm{or}\;R_{energy}^{⊤}U_{k}\;\mathrm{or}\;R_{co2}^{⊤}U_{k}\\ \text{subject}\;\text{to} & & A_{c}^{k}U_{k}\leq b_{c}^{k}\;\left(\text{linear}\;\text{bound}\;\text{constraints}\right)\\ & & B_{L}\leq U_{k}\leq B_{U}\;\left(\text{bound}\;\text{constraints}\right) \end{array}\right.$$

This algorithm is suited for joint control of TABS and shading through venetian blinds for optimised energy management. Thanks to its grey-box model, managing the complex shaded glass façade in an efficient way, this approach offers a concrete solution to implement a DMPC in a building combining TABS and shading.

As explained previously, instead of relying on weighting factors in the objective function to balance multiple objectives, this formulation handles thermal comfort as constraints. This ensures more stable and reproducible optimization results, saving computational resources and avoiding potential inconsistencies, that can arise from the manual weight adjustments. By simply exchanging the R vectors without altering the underlying model structure, the objective function can be easily adapted to various optimization tasks. For energy cost minimization, the $R_{cost}$ vector with forecasted energy prices is used. To prioritize energy demand reduction, $R_{energy}$ would be used, with unit values, giving the energy consumption. For CO_2_ emission minimization, $R_{co2}$ would be used, incorporating emission factors corresponding to the energy sources actually used.

This standardized optimisation problem formulation brings significant advantage for a flexible and efficient DMPC implementation. This enhances the model's applicability for various energy management strategies across different building systems and operational contexts.

### 7 Conclusion and perspective of this Data-driven Model Predictive Control algorithm

This article presents a reliable Mixed-Integer Linear Programming (MILP) formulation for Data-Driven Model Predictive Control (DMPC) in buildings, specifically designed to control jointly Thermally Activated Building Structures (TABS) and shading systems. Effectively managing multiple control variables, the MILP formulation maintains occupant comfort, while optimizing energy use, taking into account the physical constraints of the system. This enables the implementation of advanced control strategies that seamlessly incorporate renewable energy sources and optimize building performance for enhanced sustainability.

Compared to the previous white-box model approaches handling similar control systems, a key novelty of the algorithm presented here is that it relies on a grey-box model. This offers significant advantages for controlling buildings combining TABS and shading systems. Indeed, the method enables an easy periodic update of the grey-box model parameters, through data-driven identification. This regular calibration accounts for system changes due to wear, aging, or external influences, ensuring the model maintains its predictive accuracy over time. Additionally, this grey-box model improves the scalability of the approach, facilitating the transfer to other buildings. The implementation in different buildings would require adjustments, possibly of the grey-box model structure and probably of the façade model, but the data-driven parameter identification can be used for fast commissioning.

Additionally, the DMPC algorithm shows a convenient flexibility to implement easily various optimization goals such as minimizing energy demand, energy costs, CO_2_ emissions etc. A simple exchange of vectors in the objective function is enough, thanks to managing the thermal comfort through bound constraints, instead of as a term in the objective function (no time-consuming manual adjustments of weighting factors to balance multiple objectives).

These features enhance the model's applicability across different building types and operational contexts, making it a versatile tool for both academic researchers and industry practitioners. This integrated approach not only addresses current challenges in building energy management, but also provides a foundational framework for future research in this field.

## Method validation

As mentioned in the background section, this method has been applied successfully in a simulation study, investigating the performance of a DMPC strategy in an office building [29]. This DMPC, controlling the heating / cooling and the shading system, has been subsequently used in a real operating building, demonstrating the applicability and the performance of the approach in a long-term test [30].

Out of this experience, it has been demonstrated that the approach ensures a fast commissioning. Indeed, only two weeks of measurement data are sufficient for a reliable parameter identification of the model.

Another recommendation could be that it is not necessary to run the optimisation at every time step. Regular optimization runs can be performed hourly or at least daily, even with a shorter time-step of e.g. 15 min. These regular updates ensure a swift adjustment of the control strategies to constantly changing conditions with the unexpected real-world disturbances.

## Limitations

The grey-box model of a thermal zone has not been validated across a great variety of building types and configurations. The structure of the model should be tested and potentially adapted when applied in new buildings. Nevertheless, the implementation of the façade model in the algorithm consists in relatively little information –four parameters and a polynomial function–, which can be adapted if needed. Other venetian blinds might behave in a similar way.

The model adapts to the evolving building, through updating the parameter identification with the latest available measurement data, but there is no learning effect. Such learning capability could improve the thermal zone model through time.

The model presented controls heating and cooling, determining the requested thermal load to supply to the zone. It simultaneously controls the shading system, through the height and slat angle of venetian blinds. To control more or different HVAC systems, the method will need to be adjusted in its matrix formulation.

## CRediT author statement

**Peter Klanatsky:** methodology, software, validation, investigation, data curation, writing original draft, writing review and editing, visualisation, **François Veynandt:** writing review and editing, conceptualisation, methodology, investigation, software, **Christian Heschl:** conceptualisation, methodology, resources, writing review and editing, supervision, project administration, funding acquisition.

## Declaration of competing interest

The authors declare that they have no known competing financial interests or personal relationships that could have appeared to influence the work reported in this paper.

## Data Availability

Data will be made available on request.
